# The Influence of Carious Lesion and Bleeding Time on the Success of Partial Pulpotomy in Permanent Molars with Irreversible Pulpitis: A Prospective Study

**DOI:** 10.3390/bioengineering10060700

**Published:** 2023-06-08

**Authors:** Rami Zen Aldeen, Ossama Aljabban, Ahmad Almanadili, Saleh Alkurdi, Ammar Eid, Davide Mancino, Youssef Haikel, Naji Kharouf

**Affiliations:** 1 Department of Endodontics, Faculty of Dentistry, Damascus University, Damascus 0100, Syria; rami.zenaldeen@damascusuniversity.edu.sy (R.Z.A.); ossama.jabban@damascusuniversity.edu.sy (O.A.); ammarendo89@gmail.com (A.E.); 2 Department of Oral Pathology, Faculty of Dentistry, Damascus University, Damascus 0100, Syria; dr.manadili@gmail.com; 3 Department of Pediatric Dentistry, Faculty of Dentistry, Damascus University, Damascus 0100, Syria; salekh1889@gmail.com; 4 Department of Biomaterials and Bioengineering, INSERM UMR_S 1121, Strasbourg University, 67000 Strasbourg, France; mancino@unistra.fr; 5 Department of Endodontics, Faculty of Dental Medicine, Strasbourg University, 67000 Strasbourg, France; 6 Pôle de Médecine et Chirurgie Bucco-Dentaire, Hôpital Civil, Hôpitaux Universitaire de Strasbourg, 67000 Strasbourg, France

**Keywords:** carious lesion activity, irreversible pulpitis, partial pulpotomy, vital pulp therapy, mineral trioxide aggregate

## Abstract

This prospective study aimed to evaluate the success rate of partial pulpotomy using mineral trioxide aggregate (MTA), in permanent molars with symptomatic irreversible pulpitis. Moreover, this study aimed to investigate the effect of carious lesion depth and activity and bleeding time on the outcome of partial pulpotomy. Forty permanent molars with deep and extremely deep carious lesions clinically diagnosed with symptomatic irreversible pulpitis were included. The status of the carious lesion was evaluated clinically and radiographically to determine its activity (rapidly or slowly progressing) and depth (deep or extremely deep). A partial pulpotomy was performed and MTA was used. Clinical and radiographic analysis were performed at 3, 6 and 12 months. Chi-square analysis and Fisher’s exact test were used. Scanning electron microscope and energy dispersive X-rays were used to investigate the crystalline structures and their chemical composition onto MTA surfaces after immersion in several conditions. The partial pulpotomy was 88.9% successful, with no significant difference in outcome between deep and extremely deep carious lesions (*p* = 0.22) or between rapidly and slowly progressing lesions (*p* = 0.18). Nevertheless, all failed cases were associated with rapidly progressing lesions and extremely deep lesions. All failures occurred when the bleeding time was more than 3 min (*p* = 0.10). Different crystalline structures were detected on MTA surfaces, with higher calcium percentages in PBS conditions. Within the limitations of the present study, favorable results demonstrated that MTA might be recommended as a suitable agent for partial pulpotomy in permanent molars with irreversible pulpitis. The depth and activity of the carious lesion as well as the bleeding time are important factors in the success of partial pulpotomy treatment. The prolonged bleeding time and the extremely deep rapidly progressing caries could be related with the failure cases in partial pulpotomy treatment of irreversible pulpitis.

## 1. Introduction

Numerous chemical, thermal, microbiological and traumatic factors can cause inflammation in the dental pulp [1]. Traditionally, in the reversible stage, the tooth could be healed by the elimination of the stimulus whilst, in the irreversible stage, the pulp tissue is so damaged that it is impossible to be recovered, and thus, root canal treatment (RCT) is recommended [2].

Several clinical signs could be considered as indicators of irreversible pulpits, such as severe pre-operative pain that is spontaneous or long-lasting and is accompanied by a deep carious lesion [2]. However, several histological studies demonstrated that inflammatory changes in pulp tissues are limited to the coronal region adjacent to the carious lesion, while the residual parts of the coronal pulp tissue remain normal and uninflamed [3,4]. These histological findings, along with recent improvements in the understanding of the pulp reparative processes [5], as well as developments in the field of bioactive materials, such as calcium silicate-based materials and their biological effects [6,7,8], have made it possible to change the concept of root canal treatment and to adopt more conservative treatment strategies such as vital pulp therapy (VPT) for the management of pulpitis in mature permanent teeth [9,10].

VPT is a minimally invasive, biologically based treatment aimed at preserving the vitality of the entire dental pulp or a portion of it by sealing the pulp wound with bioactive material after removing the infected tissue [11,12]. VPT represents a group of therapeutic strategies, including indirect pulp capping, direct pulp capping, partial pulpotomy, and full pulpotomy [13]. Several clinical studies over the last two decades have demonstrated that full and partial pulpotomy could be a promising biologically based treatment alternative to RCT for the management of carious mature teeth, even with symptoms of irreversible pulpitis [14,15,16]. Nevertheless, the medical literature is divided over whether or not a partial pulpotomy could be an indication for irreversible pulpitis management in mature permanent teeth, and more clinical studies are still required [17,18].

The success of the VPT is dependent upon an accurate assessment of the inflammatory status of the dental pulp. Due to the limited link between clinical signs and symptoms and the histological state of the pulp, this assessment is, unfortunately, more predictive than accurate in clinical practice [4,19]. Therefore, to limit the predictive diagnosis in teeth presenting with a carious lesion, it may be essential to pay attention to additional criteria such as the depth of penetration and activity state of the carious lesion [20]. It has suggested that the depth and activity of the carious lesion may be an important clinical measure for the regenerative potential of the pulp tissue and the degree of pulp inflammation and may influence the outcome of pulp exposure treatment [21]. Interestingly, data on carious lesion penetration and activity concerning VPT, including partial or full pulpotomy, are seldom described in the literature. In addition, the relation between depth and activity of carious lesion, and bleeding time with the success of partial pulpotomy treatment should be clarified. Furthermore, the appearance of the pulp tissues under the amputation level after partial or full pulpotomy, as well as the time needed to control bleeding, can ensure valuable information about the degree and extent of inflammation within the pulp tissues and determine the most appropriate treatment type and prognosis [9].

Calcium silicate materials, called bioceramics, are used in dentistry, especially in endodontic treatment due to their physicochemical, mechanical and biological effects [22,23,24]. PD-MTA White (Produits dentaires, Vevey, Switzerland) is one of the calcium silicate cements which is used in dental practice due to its thin hydrophilic particles, biological activities and short setting time (15 min) [25]. However, there is no previous study in the literature which investigates the effectiveness of PD-MTA on the dental pulp in term of VPT. Moreover, the crystallographic reactions of this material after contact with saliva, blood and phosphate-buffered solution have never been analyzed.

The aim of the present prospective study was to investigate the influence of the carious lesion depth and activity as well as the bleeding time on the partial pulpotomy using PD-MTA in permanent molars with irreversible pulpitis after one year of follow-up. The null hypothesis was that there is no impact of the carious lesion depth, activity and the bleeding time on the partial pulpotomy success in permanent molars with irreversible pulpitis after one year. Moreover, it aimed to evaluate the crystallographic reaction of MTA after exposure to artificial saliva, blood and phosphate-buffered solution (PBS) by using scanning electron microscope and energy-dispersive X-rays.

## 2. Materials and Methods

### 2.1. Ethical Considerations

The study protocol was approved by the Scientific Research and Postgraduate Board of Damascus University Ethics Committee, Syria (UDDS-1819-07052018/SRC-1450). The patients signed assent and informed consent forms. Patients were offered a full pulpotomy or root canal treatment in case of treatment failure.

### 2.2. Study Design and Sample Size Calculation

The study was a prospective longitudinal single-arm clinical investigation of the predictability of partial pulpotomy with MTA in permanent molars exhibiting symptoms and signs of irreversible pulpitis. The sample size was determined using a sample-size calculation program, based on the teeth numbers included in previous studies which investigated both the outcome of full and partial pulpotomy in teeth which have reversible and irreversible pulpitis [15,26,27]. Sample size was calculated using G*Power 3.1.9.2 software (Heinrich-Heine-Universität Düsseldorf, Düsseldorf, Germany) based on a previous study [15] in order to have 95% power, an alpha error probability of 0.05 and degree of freedom at 1 which has been concluded from a total sample size of 51 teeth.

### 2.3. Participants

Sixty-four patients aged from 18 to 65 years old attending the Restorative and Endodontic Dentistry Department, Faculty of Dentistry, Damascus University were enrolled. One permanent molar per patient was included.

### 2.4. Inclusion and Exclusion Criteria

Inclusion and exclusion criteria were summarized in Table 1.

### 2.5. Patient Assessment and Operative Procedure

**Figure 1:**
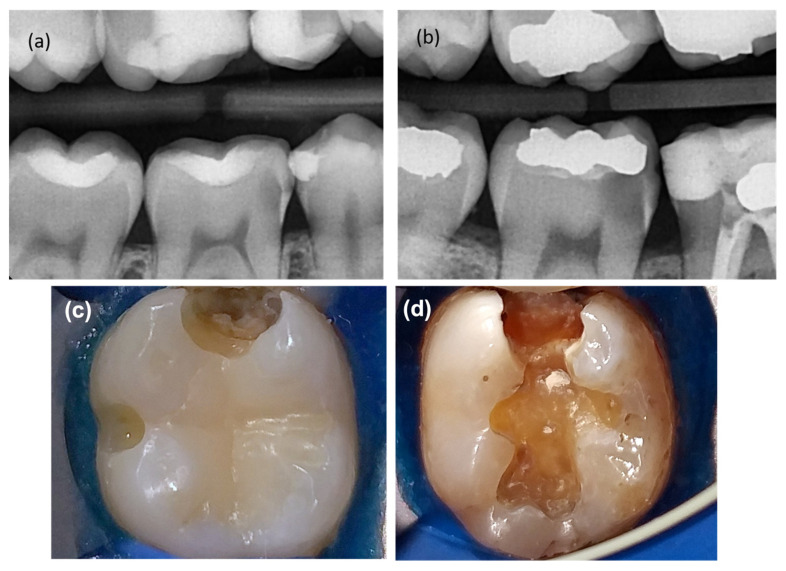
(**a**) Bitewing preoperative radiograph for first mandibular molar with deep carious lesion shows that the lesion involves the inner quarter of dentine with a radio-dense zone (hard or firm dentine) between caries and the pulp. (**b**) Bitewing preoperative radiograph for second mandibular molar with extremely deep carious lesion shows the lesion penetrating the entire thickness of the dentine with a radio-dense zone located within the pulp chamber indicative of tertiary dentine. (**c**) Intraoral image shows a rapidly progressing lesion and the yellow color of the demineralized dentine with soft and wet appearance of the surface texture. (**d**) Intraoral image shows a slowly progressing lesion and the brown color of the demineralized dentine with firm/dry appearance of the surface texture.

Before the operation, a medical/dental history and chief complaint were recorded as part of the clinical assessment. Afterward, the periodontal tissues, tooth mobility, and possibility for restoration were evaluated. In addition, the pulp response was assessed using the Endo Ice cold sensibility test (Coltene, Altstätten, Switzerland), and the pre-apical tissues were evaluated using the percussion and papulation tests. A preoperative periapical radiograph was performed using a digital sensor (Vatech, Ezsensor HD, Saul, Republic of Korea) with a film holder (Dentsply, Elgin, IL, USA) to evaluate the condition of the periapical and furcation areas. In addition, a bitewing radiograph was taken to assess the depth of penetration of the carious lesion (Figure 1a,b). The carious lesions were later divided, according to the depth of penetration within the dentine as shown radiographically, into deep (Figure 1a) (caries reaching the inner quarter of dentine but with a zone of hard or firm dentine between caries and the pulp) or extremely deep (Figure 1b) (caries penetrating the entire thickness of the dentine with certain pulp exposure) [13,28]. Later, radiographs were evaluated independently by the endodontist and a second examinator, for the depth of the lesion as a deep or extremely deep case. After that, a local anesthesia was applied using 2% lidocaine with epinephrine 1/80,000 (Scandonest; Septodont, Saint-Maur-des-fosses Cedex, France) and a rubber dam was applied. The cavity was prepared, under a dental operating microscope (Labomed, Los Angeles, CA, USA), by using a diamond bur (EX-41, Dia burs-Mani, Tochigi, Japan) with a water-cooled high-speed handpiece. Caries at the lateral walls of the cavity and only the superficial part of the demineralized dentin was removed with a sharp excavator, followed by rinsing of the cavity with saline. After drying the cavity using a sterile cotton pellet, the carious lesion was carefully examined to determine its activity. The operator assessed the level of caries activity based on the color and surface texture (moisture and consistency) of the carious dentine. The operator determined the demineralized dentine color by comparing the clinical situation with photographs of the five typical dentine color classes, which are light yellow, yellow, light brown, dark brown, or black [29]. The consistency of the dentine was categorized as soft dentine (when it can be excavated with minimum resistance using hand instruments), firm dentine (when it was resistant to excavation using manual instruments), and hard dentine (when it was resistant to probe penetration) [13]. Surface humidity was determined by inserting a probe into the carious tissue; if the tissue oozed moisture, it was categorized as wet, and if it did not, it was classified as dry [30]. Accordingly, demineralized dentin with a light yellow/yellow color and a soft/moist surface texture was classified as a rapidly progressing lesion (Figure 1c), while demineralized dentin with a light brown/brown color and a firm/dry surface texture was classified as a slowly progressing lesion (Figure 1d).

**Figure 2:**
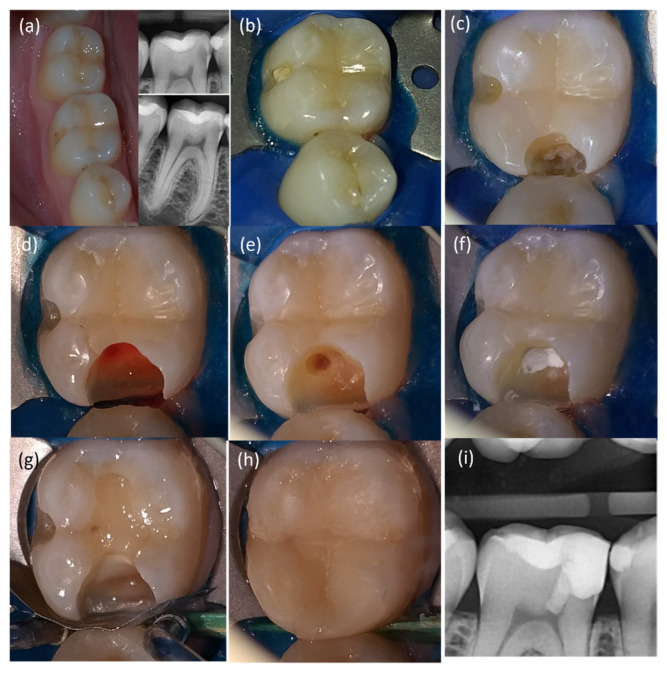
Mandibular right first molar presented with symptomatic irreversible pulpitis treated with partial pulpotomy using PD-MTA. (**a**) Clinical and radiographic examinations revealed deep carious lesion confined at a proximal surface with no evidence of dentine exposure, reflecting a close lesion environment. (**b**) Rubber dam application. (**c**) After removal of the undermined enamel and the exposition of the demineralized dentine. (**d**) Pulp exposure after non-selective caries removal. (**e**) Partial pulpotomy by removing of 2–3 mm of pulp tissues under exposure and hemostasis achieved. Note that the pulp tissues under level of amputation showed a normal appearance, texture, and color. (**f**) PD-MTA application as capping material. (**g**) Base of resin-modified glass–ionomer cement placed above MTA material. (**h**) Composite resin restoration. (**i**) Postoperative bitewing radiograph.

**Figure 3:**
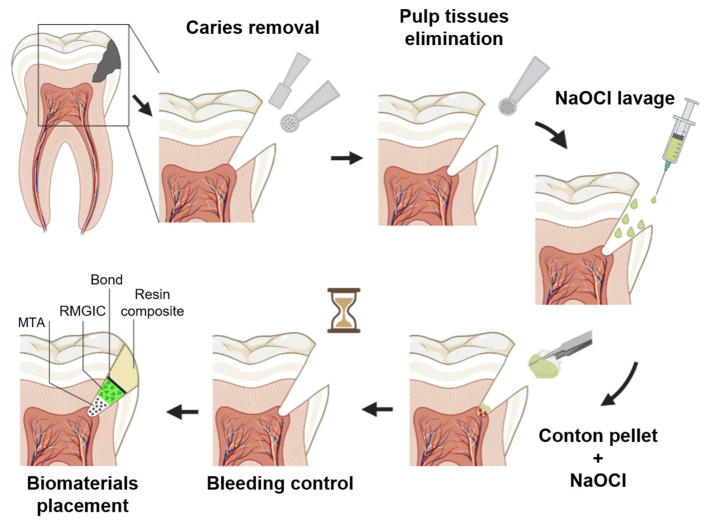
Schematic graph presents the different steps of clinical procedure in partial pulpotomy.

After that, the gross caries was removed in a non-selective manner with a round bur and a slow handpiece (Figure 2d). After the pulp was exposed (Figure 2d), a carbide bur on a high-speed handpiece was used to eliminate around 2 to 3 mm of coronal pulp tissues under water-cooling (Figure 2e). Following the rinsing of the pulp with 2.5% NaOCl [31] for one minute, the state of the pulp tissues below the amputation level was thoroughly evaluated under magnification. If the pulp tissues’ appearance was normal with no sign of infection or degradation, hemostasis was attained by applying a cotton pellet drenched in 2.5% NaOCl to the wound pulp. Hemostasis was controlled every minute for up to 6 min [26]. The time to control bleeding was recorded and subsequently divided into two categories as follows: bleeding time between 1 and 3 min, and bleeding time between 4 and 6 min. Finally, mineral trioxide aggregate (MTA) (PD-MTA, Produits Dentaires SA, Verey, Switzerland) was prepared following the manufacturer’s instructions and added gradually over the fresh pulp wound and surrounding dentine to a thickness of 2–3 mm using the MapOne carrier (MapOne system, Produits Dentaires SA, Verey, Switzerland) (Figure 2f). The MTA was coated with a sterile, moist cotton pellet immersed in saline for 15 min to provide primary hydration. After that, a layer of resin-modified glass-ionomer cement ‘’RMGIC’’ (Fuji II LC; GC Corp, Tokyo, Japan) was placed on the MTA (Figure 2g) [32]. An universal adhesive system (Tetric-N Bond, Ivoclar Vivadent) was applied and covered by direct composite restorative resin (Tetric-Ceram, Ivoclar Vivadent) (Figure 2h) [33]. All the clinical steps are summarized in Figure 3.

### 2.6. Outcome Evaluation

Three follow-up appointments (3, 6, and 12 months) were determined. At each time point, participants were clinically and radiographically examined by an endodontist who was blinded about the depth and activity of caries and the bleeding time. Different clinical parameters including the absence and presence of clinical signs, a vitality pulp test, a periodontal examination, and a percussion test were recorded. Periapical and bitewing radiographs were taken to evaluate any pathological changes at the periapical or furcal area and to detect dentinal bridge formation. The radiographs were later evaluated independently by two blinded examiners; the accuracy between the examiners was investigated by repeating the evaluation of the images after one week [34]. After one year, the teeth were classified as successful or failed treatments. To be classified as retaining overall success, the tested tooth should have both clinical and radiographic success. Treatment was considered successful when the tooth responded positively to a cold test within normal limits, there were no signs of pulpitis, there was no abnormal mobility or fistula, and there was no evidence of an apical radiolucency or internal and/or external root resorption.

### 2.7. Scanning Electron Microscope (SEM) and Energy Dispersive X-ray Analysis (EDX)

After the end of the follow-up (12 months), 9 samples of the same MTA material were prepared using Teflon molds (height: 3.8 mm/diameter: 3 mm). The samples were put at 37 °C for 48 h to achieve a good setting time [12]. Each of the three samples were stored in 50 mL of human blood, phosphate-buffered saline (PBS10x, Dominique Dutscher, Bernolsheim, France) or artificial saliva (Serlabo Technologies, Entraigues-sur-la-Sorgue, France) at 37 °C for 7 days. After the immersion time, the samples were gently rinsed with distilled water for 3 min and were sputter-coated with gold–palladium (20/80) using a Hummer JR sputtering device (Technics, San Jose, CA, USA). After that, the surface of each sample was investigated using SEM (Quanta 250 FEG scanning electron microscope “FEI Company, Eindhoven, The Netherlands”; 10 kV acceleration voltage of the electrons) and studied at a magnification of 1000× and 4000× for morphological evaluations and mineralization changes through SEM. Moreover, EDX analyses were performed for 30 s at 10 mm of distance in order to investigate the chemical composition.

### 2.8. Statistical Analyses

The SPSS 24.0 software (SPSS Science, Chicago, IL, USA) was used to perform the statistical analyses. The level of significant difference was at α = 0.05. Fisher’s exact test and the Chi-square test were used to assess the influence of the depth and activity of a carious lesion and the bleeding time on the outcome of treatment. The preoperative caries depth assessment inter-observer agreement and the postoperative intra-observer reproducibility and inter-observer agreement in terms of any pathological changes in the periapical or furcal area were assessed using Cohen’s Kappa coefficient.

## 3. Results

**Figure 4:**
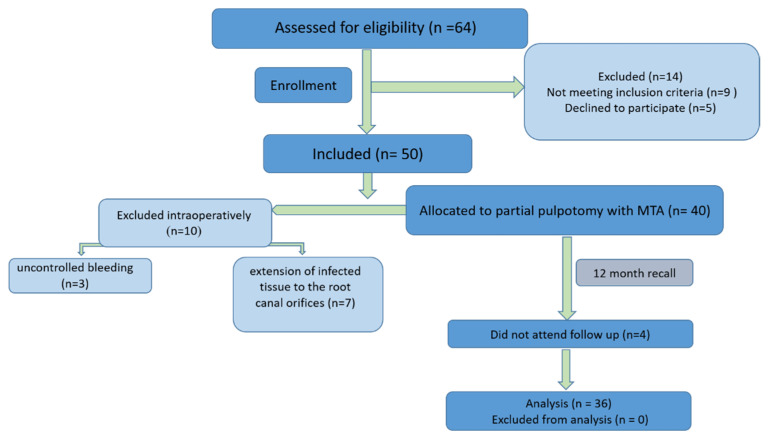
Study flow diagram.

Sixty-four patients were assessed for eligibility, having presented with signs symptomatic of irreversible pulpitis in molar teeth. Fourteen participants were excluded from the study due to refusal to participate (five patients) or not meeting inclusion criteria (nine patients). Fifty patients (50 teeth, one tooth per patient) were enrolled to be treated by partial pulpotomy treatment. Ten patients were subsequently excluded intraoperatively, three cases due to uncontrolled bleeding and seven cases due to extension of infected tissue to the root canal orifices or beyond. These patients were treated either with full pulpotomy or with RCT. Finally, forty teeth were included and treated in this study. Four participants could not attend the follow-ups, resulting in an overall recall rate of 90% (36/40) (Figure 4).

The patients consisted of 13 males and 23 females, aged 18–65 years old (32.75 ± 10.7 years old). The included cases consisted of twenty teeth (55.5%) and sixteen teeth (44.5%) with deep and extremely deep caries, respectively. The carious lesion was actively progressing in twenty-four cases (66.7%) compared to twelve slowly progressing lesions (33.3%).

The mean bleeding time was 3.80 ± 1.47 min; fifteen teeth (41.7%) needed time between 1–3 min to achieve hemostasis, while twenty-one teeth (53.3%) needed time between 4–6 min to control the bleeding.

The success rate for partial pulpotomy managing irreversible pulpitis was 88.9% after one year of follow-up, and failure was observed in four cases. Early failure occurred within three months in three cases, while late failure was observed after 12 months in one case. For carious lesion activity, all failure cases were associated with actively progressing lesions, while no failure occurred in slowly progressing lesions. However, no statistically significant difference was observed (*p* > 0.05). Regarding caries depth, only one failure case was related to a deep lesion, whilst the other three failure cases were associated with extremely deep carious lesions (*p* > 0.05).

As for bleeding time, it was noted that all failure cases were associated with a bleeding time ranging between 4 and 6 min, whilst no failure was detected for cases with 1–3 min bleeding time (*p* > 0.05) (Table 2).

The results were subdivided by both depth and activity of carious lesions into four categories: rapidly progressing deep (*n* = 14), rapidly progressing extremely deep (*n* = 10), slowly progressing deep (*n* = 6), and slowly progressing extremely deep (*n* = 6). Three cases of rapidly progressing extremely deep lesions and one case of rapidly progressing deep lesions failed, whereas neither slowly progressing deep lesions nor slowly progressing extremely deep lesions failed (*p* > 0.05) (Table 3).

The two examinations showed a good level of accordance in investigating caries depth (κ = 0.8). When assessing the periapical and furcal areas, the Kappa value for the inter-observer accordance was 0.89, and for the intra-observer reproducibility, the Kappa values were 0.95 and 0.92 for the 1st and 2nd observers, respectively.

**Figure 5:**
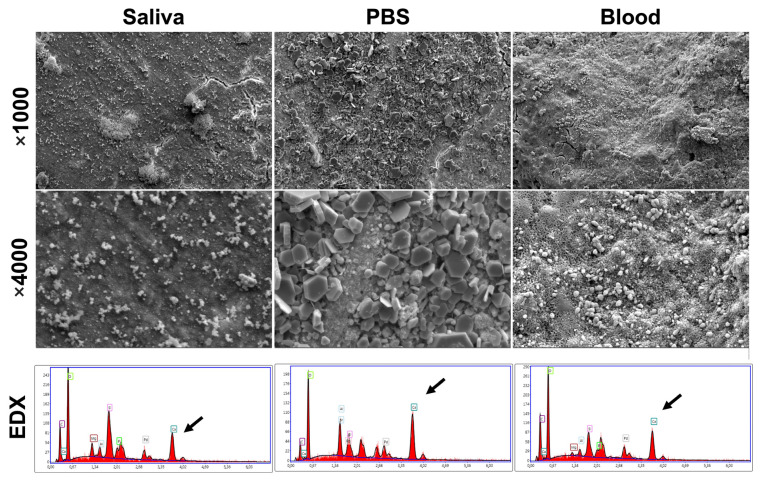
Scanning electron micrographs and Energy dispersive x-ray for Mineral Trioxide Aggregate surfaces in contact with saliva, PBS and blood at ×1000 and ×4000 magnifications. Different calcium percentages were detected by using EDX analysis (Black arrows).

SEM micrographs showed the reaction of MTA surfaces after 7 days of immersion in blood, PBS and artificial saliva at 37 °C. The crystalline textures of the MTA in the three conditions are demonstrated in Figure 5. Different crystalline appearances were detected. SEM images of MTA exposed to PBS demonstrated cubical crystalline, whilst MTA surfaces exposed to blood demonstrated small globular crystalline. MTA surfaces exposed to saliva did not show any crystalline structures. EDX analysis for MTA surfaces after 7 days presented different % of Ca, Si and P among the three conditions. Higher Ca mass percentages were detected on MTA surfaces in PBS condition compared to blood and saliva.

## 4. Discussion

Partial pulpotomy is described as the removal of 2–3 mm of pulp tissue at the exposure site, followed by sealing of the pulp wound with bioactive material [13]. This procedure differs from pulp capping in that it removes the superficial layer of infected or inflammatory tissue and the accompanying biofilms [26]. In addition, it preserves a significant portion of the coronal pulp compared to a full pulpotomy, which removes the entire coronal tissue up to the canal orifices [12]. Therefore, partial pulpotomy could be the most conservative and predictable treatment for carious pulp exposure.

The success rate in the present study was 88.9% after one year of follow-up. Several clinical studies over the last 10 years have shown success for the partial pulpotomy procedure in treating carious pulp exposure in adult and young permanent teeth, even when there are symptoms and signs of irreversible pulpitis [15,16,26]. In accordance with the results of the present study, Uesrichai et al. noted a 90% success rate for partial pulpotomy in the management of irreversible pulpitis in mature and young teeth in children aged 6 to 18 years [16]. Another study showed a success rate of 90% after 12 months partial pulpotomy procedure on complete developed permanent teeth within a sample of reversible and irreversible pulpitis [26]. A longer follow-up study (2 years) performed on partial pulpotomy in complete developed adult teeth with symptomatic irreversible pulpitis showed a slightly decrease in the success rate to 80% [15]. In accordance with the previous study of Taha and Khazali [15], the current study relied on standardizing the initial diagnosis of the dental pulp and limiting it to irreversible pulpitis in adult mature permanent molars to analyze the partial pulpotomy procedure in such cases more precisely. In addition, the originality of the current study was to associate the effect of carious lesion activity and depth, as well as the use of PD-MTA on the success of a partial pulpotomy procedure, which was never previously investigated.

No statistically significant relation was found between the failure with deep or extremely deep lesions (*p* > 0.05). Therefore, the null hypothesis must be accepted. Only one failure case was related to a deep lesion, whilst three failure cases were associated with extremely deep carious lesions. This results could be explained by the fact that pulpitis will be present at the early stage of the carious process, and the severe inflammatory response and the significant levels of inflammatory cells are not identified in the pulp until caries have progressed to within around 0.5 mm near the pulp [35]. It was found that bacteria were mainly present in the primary dentine in deep carious lesions. Extremely deep lesions, on the other hand, were linked to pulp-reaching microorganisms, as well as inflammatory infiltration and subsequent partial necrosis [20]. Therefore, the majority of failure cases in the current study were associated with extremely deep lesions rather than deep ones. In accordance, Careddu and Duncan [26] noted that all failure cases in their study, which were treated with a partial pulpotomy using Biodentine as capping material, were in the extremely-deep caries group.

Ten patients were excluded intraoperatively due to either the extension of infected tissue to the root canal orifices or beyond, as confirmed by careful examination under magnification and illumination, or uncontrolled bleeding that may indicate advanced pulpal inflammation [36]. In addition, these patients had extremely deep caries. In accordance, a previous histological study [20] revealed that more than half of extremely deep caries were associated with inflammatory infiltrates that affected the complete coronal pulp and the most coronal part of the radicular pulp, which renders partial pulpotomy inappropriate in such cases. As there was no significant difference between deep and extremely deep lesions, extremely deep carious lesions can still be treated with partial pulpotomy if a magnified examination of the underlying tissues is performed to determine the extent of inflamed and infected tissues within the dental pulp.

No failure cases were detected after one year for slowly progressing lesions, whilst all failure cases were associated with actively progressing lesions. In addition, all cases presented with slowly progressing lesions were successful even if they had extremely deep penetration, while 30% of extremely deep lesions and 7.1% of deep lesions failed if they rapidly progressed. These findings could be due to rapidly progressing lesions being associated with the presence of heavily cariogenic biofilm, while the cariogenic biofilms are significantly diminished in slowly progressing lesions [20,30]. Moreover, a slowly progressing lesion with limited cariogenic biofilms is less likely to transmit stimuli into the pulp, which is associated with a low-intensity inflammatory response favorable for healing and repair [37]. Therefore, we can conclude that the rapidly progressing and extremely deep caries associated with clinically diagnosed irreversible pulpitis have less chance of being treated with a partial pulpotomy, even though no statistically significant difference was observed in treatment outcomes according to both depth and activity of the carious lesion.

It is commonly agreed that avoiding pulpal exposure and selectively removing carious tissue is the optimal strategy for the management of a deep carious lesion [38]. In the current study, all teeth were clinically judged to have irreversible pulpitis, necessitating the total or partial removal of coronal pulp tissue [14,15]. Therefore, regardless of the depth of caries, they were chosen to have the carious tissues removed non-selectively rather than selectively. Notably, the majority of included deep lesions were proximal caries, in which the undermined enamel maintains a closed ecosystem and a rapid progression rate. These carious lesion characteristics may be associated with no or less prominent tertiary dentine formation, and bacterial invasion of the pulp may be evident even before caries reaches the pulp radiographically [39]. Moreover, pulpitis may be induced by a highly acidogenic environment in such lesions even before the pulp is exposed [28,40]. This may explain the occurrence of irreversible pulpitis in a significant part of cases included in this study despite their presenting with deep lesions without reaching the pulp radiographically, and it highlights once again the importance of evaluating the activity of the carious lesion in addition to its depth to determine the most appropriate treatment.

All failed cases in the present study occurred when the bleeding time was more than 3 min. Consistently, the present results showed no significant difference in the success rates of partial pulpotomy with the difference in the time needed for hemostasis (*p* > 0.05). In accordance, several recent studies demonstrated that there was no marked association between bleeding time and the outcome of pulpotomy treatments [18,26]. The control of bleeding is essential for the success of VPT, as the profuse, difficult-to-stop pulp bleeding could be an indicator of advanced pulp inflammation [36]. However, the mechanical and chemical elimination of the infectious challenge at the pulp wound by partial pulpotomy and NaOCl lavage may make the bleeding time a less significant factor in the treatment outcome [31]. Moreover, the local anesthesia which was applied using 2% lidocaine with epinephrine 1/80,000 in the present study could play an important role on the bleeding time. Chu et al. demonstrated that the use of local anesthesia based on lidocaine and epinephrine decreases the pulpal blood flow and may protect the dental pulp by attenuating the increase in pulpal blood flow caused by tooth preparation [41]. Moreover, it is known that the infiltration anesthesia is used successfully in the maxillary teeth but is less effective in the mandibular molar regions due to the density of bone [42]; therefore, block anesthesia was used in the present study for the mandibular molar regions.

In order to clarify the outcomes of the use of MTA in partial pulpotomy, when this biomaterial is in direct contact with pulpal tissue, blood and dentinal fluids, SEM analyses were performed on a MTA surface after immersion in PBS, blood and artificial saliva at 37 °C for 7 days. PBS was utilized to simulate the in vivo dental tissue fluids [43] to assess the mineral development that could take place on the material surfaces. SEM images showed crystallite formation on the material surfaces in the cases of PBS and blood conditions, whilst no mineralization process and no notable changes were demonstrated for MTA surfaces after immersion for 7 days in artificial saliva. Therefore, MTA is a bioactive material which could promote the remineralization process. In addition, calcium silicate-based materials are capable of making calcium hydroxide and calcium silicate hydrate when it is in a humid medium [22,24,44]. Lower calcium peaks were found for the MTA surfaces immersed in blood and artificial saliva, whilst higher calcium peeks were detected for MTA surfaces exposed to PBS. These findings could be related to the different environmental conditions and the dissolution of calcium hydroxide because of hydration [45]. These outcomes are similar to the results of a previous study which was conducted on MTA ProRoot [45]. Moreover, in the present study, RMGIC was used on MTA in order to have the optimal bond strength with the final restoration (composite) as described previously [46,47]. In addition, several studies have also shown that the MTA’s physical properties might be affected by the acidic environment that existed before composite buildup [48,49]. Therefore, RMDIC was used to have an optimal coronal sealing ability, which is related to the long success of VPT.

Some limitations were detected in the present study. For example, because the study was designed to investigate the effect of the depth and activity of the carious lesion, there was no control group to which the patients could be assigned randomly. Moreover, PD-MTA as a powder–liquid bioceramic material should be carefully mixed following the manufacturer’s instructions in order to avoid errors during manual mixing, which could alter the physicochemical properties of this material [22]. Moreover, the irrigant which was used, NaOCl, is known for its high cytoxicity, which could lead to severe tissue damage if it is used in high concentrations [50,51]; in contrast, 2.5% NaOCl has a high efficacity as a lavage solution due to the unique capability of NaOCl to selectively dissolve necrotic soft tissue, thus reducing the necessity to mechanically remove infected tissues [31]. In addition, radiographic monitoring was crucial to follow the treatment results; however, the indication to take radiographs in clinical practice is limited to reasonable requests in order to decrease patients’ exposure to ionizing radiation [52]. Further studies with higher numbers of included cases and longer follow-up periods should be performed to investigate the relation between the activity of a carious lesion and the success of partial pulpotomy treatment. In addition, the carious lesion depth was estimated on a bitewing radiograph. Although there was a good inter-observer agreement (0.8), it was still challenging to identify the exact depth of caries on a two-dimensional radiograph. Finally, future studies should consider using cone-beam computed tomography (CBCT) as a more precise technique for this purpose. The color, moisture, and surface texture of the carious tissues were also used as indications of carious lesion activity. These indicators, however, are somewhat subjective, and a more reliable method is required.

## 5. Conclusions

Within the limitations of the present study, favorable results demonstrated that MTA might be recommended as suitable agent for partial pulpotomy in permanent molars with irreversible pulpitis, with an 88.9% success rate after one year. It could be concluded that the depth and activity of the carious lesion as well as the bleeding time are important factors in the success of partial pulpotomy treatment. The prolonged bleeding time and the rapidly progressing extremely deep lesions could be related to the failure cases in partial pulpotomy treatment of irreversible pulpitis.

## Figures and Tables

**Table 1:** Inclusion and exclusion criteria.

Inclusion Criteria	Exclusion Criteria
○ The patient should be ≥18 years old. ○ Mature first/second (upper/lower) molar tooth. ○ Clinical examination shows: Tooth with a history of signs of irreversible pulpitis such as spontaneous pain or pain exacerbated by cold stimuli and lasting for a few seconds to several hours (interpreted as lingering pain) compared to control teeth, and which could be reproduced using cold testing. Positive response to cold with no signs of pulpal necrosis including swelling or sinus tract. The tooth is restorable with no need for crown or post-retained restoration. Normal probing pocket depth and tooth mobility ○ Radiographic examination shows: Mature molar with deep or extremely deep carious lesion. A pulp chamber of relatively normal dimensions without calcified forms (e.g., pulp stone, diffuse calcification, disk-like chamber) No prominent radiolucency at the furcation or zones. No evidence of internal or external resorption.	○ Immature molar with open apex. ○ Radiolucency at the periapical zones or furcation ○ Existence of calcification and internal or external resorption ○ Existence of swelling of sinus tract and the negative response to cold indicator ○ Uncontrolled bleeding after partial pulpotomy (> 6 min) ○ Bleeding is not sufficient after pulp exposure; the pulp is judged partially necrotic. ○ Non-restorable teeth.

**Table 2:** Fisher’s exact test results to compare the outcome according to the activity of carious lesion, carious lesion depth, and bleeding time.

Variables			Overall Outcome		*p*-Value
	*n* (%)		Success (%)	Failure (%)	
Activity of carious lesion	Rapid progression	24 (66.7)	20 (83.3)	4 (16.7)	0.18
	Slow progression	12 (33.3)	12 (100)	0 (0)	
Carious lesion depth	Deep	20 (55.5)	19 (95)	1 (5)	0.221
	Extremely deep	16 (44.5)	13 (81.3)	3 (18.7)	
Bleeding time	1–3 min	15 (41.7)	15 (100)	0 (0)	0.102
	4–6 min	21 (58.3)	17 (81)	4 (19)	

**Table 3:** Chi-Square test results to compare the outcome according to depth/activity of carious lesion.

		Overall Outcome		
State of Carious Lesion	*n* (%)	Success (%)	Failure (%)	*p*-Value
Rapidly progressing deep	14 (38.88)	13 (92.9)	1 (7.1)	0.149
Rapidly progressing, extremely deep	10 (27.8)	7 (70)	3 (30)	
Slowly progressing, deep	6 (16.66)	6 (100)	0(0)	
Slowly progressing, extremely deep	6 (16.66)	6 (100)	0(0)	

## Data Availability

Data are available from the first author (ramizen989@gmail.com (R.Z.A.)).
